# Survey-based data on food security, nutrition and agricultural production shocks among rural farming households in northern Uganda

**DOI:** 10.1016/j.dib.2019.103818

**Published:** 2019-03-12

**Authors:** Chris Miyinzi Mwungu, Kelvin Mashisia Shikuku, Christopher Atibo, Caroline Mwongera

**Affiliations:** a International Centre for Tropical Agriculture (CIAT), Nairobi, Kenya; b World Fish, Penang, Malaysia; c Gulu University, Uganda

**Keywords:** Resilience, Nutrition, Food security, Shocks, Uganda

## Abstract

Climate change, degradation of natural resources, conflict or civil war, diseases and poverty are among the key threats that impact agriculture, human nutrition, food security and food safety among rural households in developing countries. Sustainability of food systems and livelihoods will thus crucially depend on not only the ability to accommodate or recover from these threats but also to tap into opportunities for strengthening long-term capabilities. One approach to enhancing resilience to enhance food security and nutrition is building an evidence base to better understand the various types of smallholders, threats to agriculture production and the associated risks to food security and nutrition and household food preferences. Unfortunately, such data in many African countries is still unavailable or has not been shared publicly. In this paper, we describe data that were collected in Nwoya district, Northern Uganda in December 2017. These data can be used to assess the relationship between resilience of farm households to climatic risks and their food and nutrition security.

**Table:** Specifications table

Subject area	Agricultural Economics, Food, and Nutrition security, Climate Science
More specific subject area	Resilience, Food security
Type of data	STATA and excel files
How data was acquired	Survey
Data format	Cleaned raw data
Experimental factors	Face to face interviews were conducted using Census and Survey Processing System (CSPro) on tablets through a survey.
Experimental features	Data were collected from randomly selected households that had participated in our previous studies in Nwoya district.
Data source location	Nwoya district in northern Uganda.
Data accessibility	Available at:https://dataverse.harvard.edu/dataset.xhtml?persistentId=doi:10.7910/DVN/HPMKAW
Related research article	Shikuku, K., Mwongera, C., Winowiecki, L. A., Twyman, J., Atibo, C., & Läderach, P. (2015). Understanding farmers' indicators in climate-smart agriculture prioritization in Nwoya District, Northern Uganda.

**Table:** 

**Value of the data** • The data can allow researchers to understand the agricultural production shocks currently facing the households, perceived impacts on food security, livelihoods and coping strategies being implemented; • The data allow researchers to estimate resilience of farm households to climate threats; • Building an evidence base to better understand the driving factors beyond the various agricultural production threats, the associated risks they pose to food security and nutrition; • These data can also help us to understand how household demographics and gender relate to food security and resilience.

## 1 Data

This paper presents data that were collected in December 2017 to assess the resilience of farmers to climate change. The questionnaire was divided into 7 modules. Module A focused on the geographical location of the farmer and when the interview was conducted. Module B entailed asking farmers if they had experienced any shocks for the last five years that affected them economically. In Module C, farmers were asked questions on market accessibility. Module D, which was one of the core models in this survey, focused on household food basket and food expenditure while module E was all about nonfood household expenditures. In Module F, we asked farmers typical nutritional questions that can be used in the computation of Household Food Insecurity Access Scale (HFIAS) for measurement of food access. Lastly, in Module G, data on food scarcity and seasonality was collected.

## 2 Experimental design, materials and methods

### 2.1 Data collection site

These data were collected in Nwoya district. The district has an average annual temperature of 23 °C and two rainy seasons. The first rainy season is received from March to June while the second rainy season is revived from July to November. The mean annual rainfall received in the district is about 1,500 mm. Subsistence farming is the main economic activity in the region [1].

The main purpose of collecting these data was to link between agricultural production, shocks, food security and nutrition. The primary research questions were:

• What is the food security situation in Northern Uganda?
• What are the key driving factors affecting resilience, food security and nutrition in northern Uganda?

The ideas behind the questionnaire design were based on the above research questions and our previous surveys and research results in Nwoya district [2], [3], [4], [5].

### 2.2 Sampling strategy

While collecting this dataset, four sub counties in Nwoya district were selected excluding the town council. Then a list of all parishes, villages and sub villages were generated including the names of the household heads and their spouses. A random sample of 322 was randomly drawn from a total of 1036 households. However, for this data collection activity the person of interest that we wanted to talk to was the wife to the head of the household or any other adult who usually prepares food in the household, since they were in better position to respond the questions.

### 2.3 Survey implementation

The data were collected with the assistance of experienced enumerators who had participated in several previous surveys conducted by the International Center for Tropical Agriculture (CIAT) in Nwoya district. The enumerators were selected based on their performance from the previous surveys. They received a three-day training on the questionnaire, use of CS-Entry to collect data on android based tablets and professional ethics on collecting socio-economic data. During the training, the questionnaire was also translated in the local language to make it easier for the enumerators while asking questions. The questionnaire was piloted in Amuru, Lamogi parish of Gulu district and later adjusted, especially on the CSPro dictionary and logics. The field coordinator identified local leaders in each village who introduced the enumerators to each household prior to the interview. Overall the survey dates were from 3rd to 19th December 2017.

### 2.4 Data management and code availability

During the survey a research associate from CIAT checked for inconsistencies, patterns and outliers in the data, and later briefed the enumerators each day. The data collected in this survey were imported into STATA for cleaning. The do file that was used to clean raw data has been shared online at: https://github.com/miyinzi/resilience_data .

**Fig. 1:**
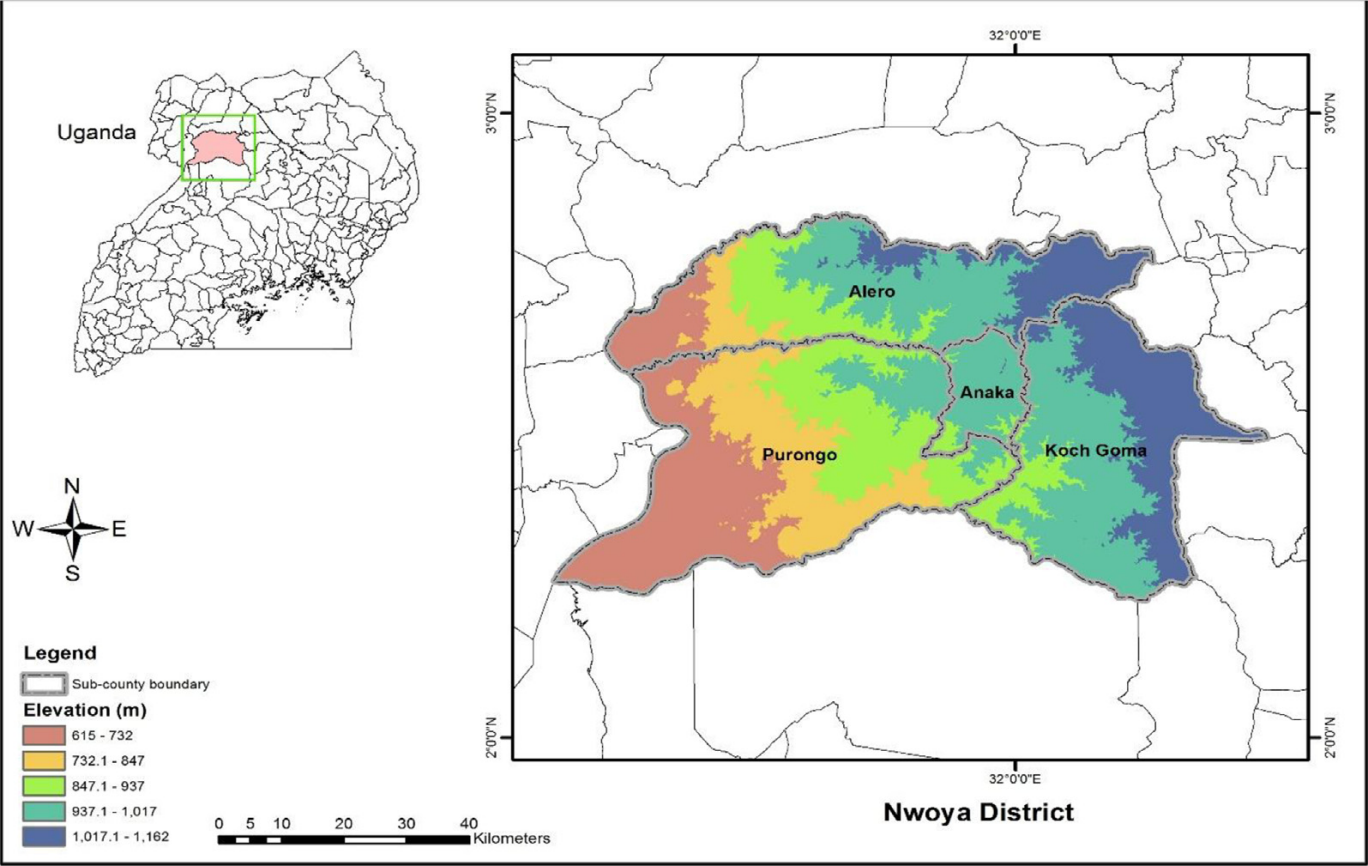
Map of Nwoya district.

### Funding sources

This work was carried out by the International Center for Tropical Agriculture (CIAT) as part of the CGIAR Research Program on Climate Change, Agriculture and Food Security (CCAFS). The project, Increasing Food Security and Farming System Resilience in East Africa through Wide-Scale Adoption of Climate-Smart Agricultural Practices, was funded with support from the International Fund for Agricultural Development Grant number 2000000176**.**
